# Relationship between *Ganoderma* Ergosterol Concentration and Basal Stem Rot Disease Progress on *Elaeis guineensis*

**DOI:** 10.21315/tlsr2020.31.1.2

**Published:** 2020-04-07

**Authors:** Muniroh Md Saad, Nusaibah Syd Ali, Sariah Meon

**Affiliations:** Faculty of Agriculture, Universiti Putra Malaysia, 43400 UPM Serdang, Selangor, Malaysia

**Keywords:** *Ganoderma*, Basal stem rot, HPLC, Microwave assisted extraction, Thin layer chromatography (TLC), Oil palm, *Ganoderma*, Reput pangkal batang, HPLC, Pengekstrakan berbantukan ketuhar gelombang mikro, TLC, Sawit

## Abstract

Basal stem rot (BSR) is a devastating disease to Malaysian oil palm. Current techniques employed for BSR disease detection on oil palm are laborious, time consuming, costly, and subjected to accuracy limitations. An ergosterol detection method was developed, whereby it correlated well with the degree of infection in oil palm. This current study was designed to study the relationship between *Ganoderma* biomass, ergosterol concentration, BSR disease progress and to validate the efficiency of microwave assisted extraction (MAE) method for extraction of ergosterol compound. In addition, testing on the sensitivity of thin layer chromatography (TLC) analysis for detection of ergosterol was also the aim of this study. The optimised procedure involved extracting a small amount of *Ganoderma*-infected oil palm root tissues suspended in low volumes of solvent followed by irradiation in a conventional microwave oven at 70°C and medium high power for 30 s, resulting in simultaneous extraction and saponification. Based on the results obtained, MAE method may be effective in extracting low to high yields of ergosterol from infected oil palm roots demonstrating disease scale 2, 3 and 4. Positive relationship was observed between ergosterol content and inoculation period starting day 3 in the inoculated oil palm seedlings and hour 6 in germinated seeds. TLC analysis demonstrated a good correlation with high performance liquid chromatography (HPLC) quantification. Therefore, a semi-quantitative TLC analysis may be applied for handling a large amount of samples during onset field survey.

## Abstract (ms)

Reput pangkal batang (BSR) adalah penyakit yang membawa kemusnahan kepada industri sawit di Malaysia. Teknik semasa yang digunakan bagi mengesan penyakit BSR pada sawit adalah sukar, memakan masa, mahal, serta mempamerkan kadar ketepatan yang terbatas. Oleh itu, kaedah pengesanan ergosterol patogen telah dibangunkan, di mana ia berkorelasi baik dengan tahap jangkitan pada sawit. Kajian ini direka bagi mengkaji hubungan di antara jisim *Ganoderma*, kepekatan ergosterol, perkembangan penyakit BSR dan untuk mengesahkan kecekapan kaedah pengekstrakan berbantukan ketuhar gelombang mikro (MAE). Di samping itu, ujian terhadap kepekaan analisis plat kromatografi lapisan nipis (TLC) bagi pengesanan ergosterol juga merupakan tujuan kajian ini. Prosedur yang telah dioptimumkan melibatkan pengekstrakan sejumlah kecil tisu akar sawit yang telah dijangkiti patogen *Ganoderma* yang diampai dalam pelarut dengan isipadu rendah. Ini diikuti oleh penyinaran dalam ketuhar gelombang mikro konvensional pada suhu 70°C dan kuasa sederhana tinggi selama 30 s, menghasilkan pengekstrakan dan saponifikasi secara serentak. Berdasarkan keputusan yang diperolehi, kaedah MAE mungkin berkesan dalam mengekstrak ergosterol daripada kuantiti tisu sawit yang sedikit tetapi mampu menunjukkan tahap penyakit sawit dari skala 2, 3 dan 4. Hubungan positif diperhatikan di antara kandungan ergosterol dan tempoh inokulasi bermula hari ke-3 dalam benih sawit yang telah diinokulasi dan 6 jam selepas inokulasi bagi benih yang telah bercambah. Analisis TLC telah menunjukkan korelasi yang baik dengan kuantiti kromatografi cecair prestasi tinggi (HPLC). Oleh itu, analisis separa kuantitatif TLC boleh digunakan untuk mengendalikan sejumlah besar sampel semasa tinjauan di lapangan.

**Highlights**

- Ergosterol was detected as early as six hours and three days after inoculation with oil palm’s germinated seeds and seedlings respectively.
- The concentration of ergosterol increased with the inoculation period and disease severity.
- For infected field palm, ergosterol was detected from all sample categorised in scale 2, scale 3 and scale 4 and absent in scale 1 palms.

## INTRODUCTION

Oil palm (*Elaeis guineensis*) is a monocotyledon in the family Arecaceae (formerly Palmae) within the subfamily Cocosoideae (Corley & Tinker 2003). It is a major crop in the tropical areas, particularly in the Southeast Asia. Palm oil is used worldwide for the production of food products, cosmetics, pharmaceuticals, biodiesel and in oleochemical industry (Kalam & Masjuki 2002; Corley & Tinker 2003; Turner *et al*. 2008). Oil palm industry contributes to the Malaysian economy by triggering the development of country’s rural areas (Chin 2008). In Malaysia, cultivation of oil palm has increased year by year with 1.5 million ha in 1985 to 5.84 million ha in 2018 (Malaysian Palm Oil Board 2018).

Oil palm is subjected to numerous devastating diseases such as basal stem rot (BSR), vascular wilt, spear rot-bud rot, sudden wither and red ring (Corley & Tinker 2003). However, BSR is the major disease encountered by Malaysian palms, which is caused by *Ganoderma* spp. (Idris *et al*. 2011). Paterson (2019) reported that the disease is increasing in inland Peninsular Malaysia and also Sabah, Malaysia, and in some cases at high levels, whereas it has not been detected before. Several attempts have been made to control BSR using various control methods; however to date, none of the methods gave promising results in management of *Ganoderma boninense,* the major causal pathogen of BSR disease (Ariffin *et al*. 2000; Sanderson *et al*. 2000; Susanto *et al*. 2005). The exertion of handling this disease is due to the infected palms not showing any external symptoms on mature palms until advanced stage. When it comes to this stage, the infected trees may not be able to respond to any treatment given (Bivi *et al*. 2016). At present, the most common way used to detect BSR is based on the foliar symptoms and appearance of basidiomata at the base of infected stem. However, by the time visible symptoms appear, the palms were already at the final stages of infection and usually half of the basal tissues have been killed by the fungus (Idris 2009). An early detection of BSR disease could prolong the economic life span of a palm (Lim *et al*. 1993). Therefore, enzyme-linked immune sorbent assays-polyclonal antibody (Idris & Rafidah 2008) as well as polymerase chain reaction (PCR) based techniques involving *Ganoderma-*specific primers (Bridge *et al*. 2000; Utomo & Niepold 2000; Yamoaka *et al.* 2000) have been proposed as early detection methods of the disease. However, these methods are complicated and time consuming for early detection of the disease in oil palm fields. Moreover, there are some limitations with PCR technique that requires to be addressed before applying for detection of *Ganoderma* (Paterson 2007a; Paterson *et al*. 2008; Paterson & Lima 2009). For instance, PCR could be subjected to inhibition (Paterson 2007a) and ELISA-PAB (ELISA) suffers from cross reactivity (Idris & Rafidah 2008). Hence, a feasible early detection method of the disease is required and crucial to prolong palm’s life span via available curative methods.

Ergosterol is part of cell wall component of a fungus which is exclusively found in higher fungi and absent in other organisms (Madonna *et al*. 2001; Mille-Lindblom *et al*. 2006). It is essential to fungus and its absence results in the death of the fungus (Morpurgo *et al.* 1964) thus indicating live fungal biomasses. The detection of ergosterol as fungal biomarker is measured to be the method of preference (Parsi & Górecki 2006). Ergosterol has been successfully used to indicate fungal biomass in soil (Grant & West 1986; Frostegard & Baath 1996), pathogenic fungi in roots and cereal grains (Bindler *et al*. 1988; Seitz *et al*. 1977), saprophytic fungi in decaying plant materials (Newell *et al*. 1988), ectomycorrhizal fungi in roots and soil (Salmanowicz & Nylund 1988; Wallander *et al*. 1997), and recently in oil palm tissues (Mohd Aswad *et al*. 2011; Toh Choon *et al*. 2012; Chong *et al*. 2014; Bivi *et al*. 2016). Several attempts have been made to determine the relationship of ergosterol and fungal biomass under various conditions (Newell 1992).

First data published on the use of ergosterol analysis as a diagnostic method to detect BSR supports the view that ergosterol exhibits the effectiveness for detection of BSR in oil palm (Mohd Aswad *et al*. 2011). Parkinson and Coleman (1991) reported that ergosterol assay was commonly considered the most promising tool for detection and quantification of fungal biomass. Parsi and Górecki (2006) also reported that the detection of ergosterol as fungal biomarker could be considered as the method of choice.

Previous studies applied organic solvent-based methods such as non-alkaline extraction (NAE) (Mohd Aswad *et al*. 2011), alkaline extraction (AE) (Zill *et al*. 1988), and ultrasonication extraction (USE) (Yuan *et al*. 2007) methods for extraction of ergosterol. These organic solvent-based methods (conventional) typically requires large samples size, large reagent volume, it is labour intensive and time consuming, additionally AE and USE were reported to yield low concentration of ergosterol compared to NAE method (Mohd Aswad *et al*. 2011). Therefore, an efficient extraction method is required for the extraction of ergosterol. Young (1995) has developed MAE method for ergosterol extraction which requires a smaller sample size and reagent volume, it is more economical in terms of chemical used, and using convectional equipment (domestic microwave). MAE is therefore more convenient than other methods in terms of time for sample preparation, cost and sample size. In addition, a larger sample size could be extracted at one time; moreover, the method is simple, rapid and reliable for ergosterol detection on palms in the field environment during census carried out on disease survey.

Hence, the present study was undertaken to establish relationship between *Ganoderma* biomass, ergosterol concentration and BSR disease progress in germinated seeds, artificially inoculated oil palm seedlings, and infected oil palm field tissues. Additionally, validation on the efficiency of MAE method for extraction of ergosterol and to test the sensitivity of thin layer chromatography (TLC) analysis for detection of ergosterol in artificially inoculated germinated seeds, artificially inoculated oil palm seedlings and infected oil palm field tissues was another objective.

## MATERIALS AND METHODS

### Mycelial Culture of *Ganoderma*

A pure culture of *G. boninense* was isolated from a basidiomata of an infected oil palm trunk in Felda Gua Musang, Malaysia using *Ganoderma* selective medium (GSM) (Ariffin & Idris 1991).

### Molecular Identification

Molecular identification was conducted to confirm the *Ganoderma* culture. DNA was extracted using the modified CTAB method of Doyle and Doyle (1987). PCR amplification was done as described by Utomo and Niepold (2000) with some modification on annealing temperature and amplification cycle. The PCR mixture containing 12.5 μL of Ampoun PCR master mix, 1 μL of both forward and reverse *Ganoderma* specific primers (Gan 1: 5’ TTG ACT GGG TTG TAG CTG 3’ and Gan 2: 5’ GCG TTA CAT CGC AAT ACA 3’) (Utomo & Niepold 2000) and 9.5 μL of nucleus free water were prepared in a 24 μL reaction volume. Then, 1 μL of DNA template was added to a final volume of 25 μL. The thermo cycler was programmed as follows: 5 min at 95°C, 35 cycles of 35 s at 94°C, 35 s at 59.2°C, 40 s at 72°C, and 10 min at 72°C. The PCR products were analysed by electrophoresis on a 1.5% agarose gel and stained with ethidium bromide to visualise the amplicates under UV light. The molecular identification from PCR product were sequenced using DNA sequencing services (Apical Scientific Sdn. Bhd., Malaysia) and aligned using Basic Local Alignment Search Tool (BLAST) network services against National Centre for Biotechnology Information (NCBI).

### Quantification of Ergosterol

#### Ergosterol extraction

Extraction of ergosterol from roots tissue were carried out using MAE method based on the procedure by Muniroh *et al.* (2014). 1.0 g of oil palm’s root tissue was macerated in liquid nitrogen using a mortar and pestle into a powder, and transferred to a Pyrex test tube with a Teflon screw cap. 2 mL of methanol (Chromatography grade, Merck, United State) and 0.5 mL of 2M sodium hydroxide was added and the tube was tightly sealed. The test tubes were placed in a culture jar at the centre of a conventional microwave (Sharp Jet Convectional Grill, model TTAG A437 with capacity 1.5 cu. ft, Sharp, Japan) and subjected to microwave setting of 70°C, and medium high power with 30 s exposure time. The solutions were left to cool and were neutralised with concentrated hydrochloric acid. Finally, the solutions were extracted three times with 2 mL of pentane (Fisher chemicals, analytical reagent grade). The combine pentane extracts were then evaporated to dryness by using a Buchi Rotary Evaporator (Buchi, Switzerland) and then dissolved in 500 μL methanol for detection of ergosterol using TLC and quantified using HPLC with an ergosterol standard (Sigma, purity ≥ 95.0%, Sigma-Aldrich, Germany).

#### Semi quantitative TLC

TLC was carried out to detect the presence of ergosterol from the extracted root tissues. TLC detection was undertaken based on Mohd Aswad *et al.* (2011) in duplicate for all samples. The *R_f_* value was calculated using this formula:

$$R_{f}=\frac{\text{Distance travelled by the product}}{\text{Total distance travelled by the solvent}}$$

#### High performance liquid chromathography (HPLC)

An Agilent 1100 series HPLC equipped with a Diode Array Detector (G1315B), a pump (G1311A), and an auto sampler (G1313A) was used for quantification of ergosterol using an Ascentis express 2.7 μ C18 reverse-phase column (Supelco, USA). Operating conditions was based on Mohd Aswad *et al.* (2011).

#### Germinated Oil Palm Seeds

Germinated seeds (Dura × Pisifera) used were supplied by Sime Darby Research Centre, Banting, Selangor. They were maintained in sterilised sand containing Hoagland solution for two weeks to allow rooting.

The experimental design for this experiment was complete randomised design (CRD) consists of two experimental treatments: Non-inoculated with *G. boninense* (T1) and inoculated with *G. boninense* (T2). The germinated seeds were uprooted carefully and rinsed with distilled water. Germinated seeds for treatments (T2) were placed into 45-culture jar containing MAE slant culture of *G. boninense* (isolated from Gua Musang Felda) with three roots per culture jar. Non-inoculated germinated seeds (culture jar containing only MEA) were used as negative control. Random samplings of the experimental materials were done over a period of 6, 12, 24, 48, 72, 96, 120, 144 and 168 hours after inoculation with five culture jars per sampling time. All the roots were pooled and subjected to detection and quantification of ergosterol and further confirmed with PCR using modified method by Utomo and Niepold (2000). The root samples from 0, 6, 24 and 48 hours were also subjected to Scanning Electron Microscope (SEM) (in-house method, Microscope Unit, Institute of Bioscience, Universiti Putra Malaysia) to view the physiological changes after inoculation.

### Oil Palm Seedling

The experiment was repeated using six month old oil palm seedling (Dura × Pisifera) supplied by Sime Darby Research Centre, Banting, Selangor. The seedlings were maintained in polybags in the glasshouse until five to six leaf stages. The seedlings were watered daily and fertilised with NPK fertiliser (10 g per polybag) at monthly interval.

Eighty of 6 months-old oil palm seedlings were used for the infection study conducted in a glasshouse with two experimental treatments; non-inoculated with *G. boninense* colonised rubber wood block (RWB) (T1) and inoculated with *G. boninense* colonised RWB (T2). The seedlings were uprooted carefully and transplanted into polybags (size 12 cm × 15 cm) containing 3 kg soil mixture (3:2:1 v/v/v topsoil: peat: sand). Treatment (T2) was inoculated with a *G. boninense* mycelium colonised RWBs placed in contact with the roots (Sariah *et al*. 1994). Non-inoculated seedlings were used as negative control. All oil palm seedlings were placed and arranged in a randomised complete block design (RCBD) under glasshouse conditions for 28 weeks. The seedlings were watered twice daily. Random destructive sampling of the seedlings was carried out on day 3, week 1, week 2, week 4, week 8, week 12, week 16, week 20, week 24 and week 28 with five replicates for each sampling. The root samples were used for detection and quantification of ergosterol.

A visual assessment of BSR infection was determined by examining the roots and foliar symptoms of the seedlings. The seedlings were also split longitudinally to observe root and bole decay and to visually assess the severity of the symptoms based on the proportion of number of lesion (rotting) roots. The estimation was based on the scale modified from Breton *et al.* (2005) (Table 1)**.** Disease severity (DS) for internal and external symptoms of roots tissues and foliar symptoms was calculated based on formula derived from Liu *et al*. (1995) as follows:

$$\begin{array}{l} \text{DS }(\text{Internal})=\frac{\sum \;\text{Number of seedlings in the rating}\times \text{rating number}}{\text{Total number of seedlings assessed }\times \text{highest rating}}\times 100\\ \text{DS }(\text{External})=\frac{\Sigma \;\text{Number of seedlings in the rating}\times \text{rating number}}{\text{Total number of seedlings assessed }\times \text{highest rating}}\times 100 \end{array}$$

### Infected Oil Palm Field Tissues

Oil palm tissue samples were collected from high BSR disease incidence plot at Serting Felda Plantation Berhad, Negeri Sembilan. Mature palms aged 13 years old were randomly chosen based on the appearances of external symptoms of BSR disease and were categorised into scale 1, scale 2, scale 3 and scale 4 with 15 palms for each category (Table 2). Tissue samples (1.0 g) were weighed and grinded using liquid N_2_ in a mortar and a pestle into fine powder. Samples were subjected to MAE extraction, TLC and HPLC analysis.

## STATISTICAL ANALYSIS

The data were analysed using SAS Release 6 (SAS Institute Inc. 1990). Triplicate determinations of ergosterol concentrations from each sample were analysed using ANOVA and means were compared by Least Significant Difference (LSD) (*P* ≤ 0.05). Correlation analysis was performed using Microsoft Excel 2007.

## RESULTS

### Identification of Mycelial Culture of *Ganoderma*

**Figure 1:**
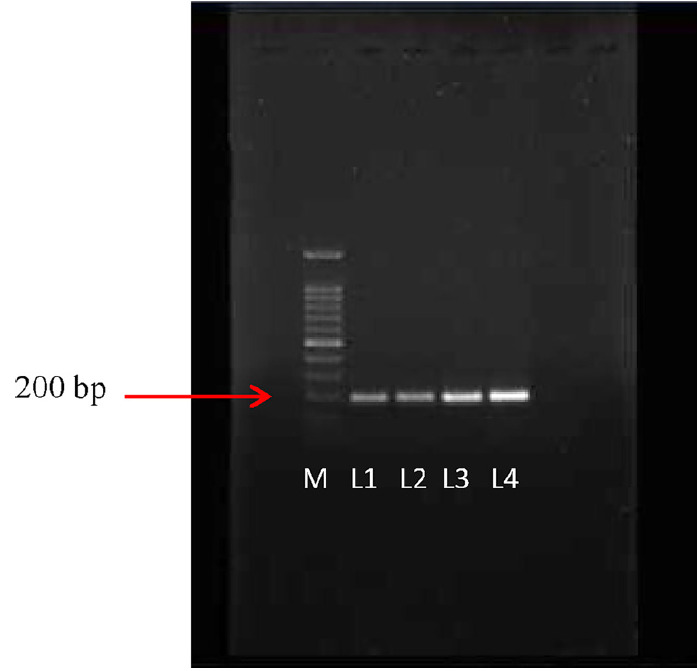
PCR amplification of 14 days old *Ganoderma* mycelial culture. M: Marker; L1–L4: amplified band of 14 days old *Ganoderma* mycelial culture.

Nucleic acid of *Ganoderma* mycelium extracted using CTAB method was further identified using molecular identification. PCR amplification with *Ganoderma* specific primer Gan1 and Gan2 were analysed with 1.5% agarose followed by ethidium bromide staining showed visible band on the expected region at 150–200 bp (Fig. 1). Gene bank database confirmed the samples were highly similar to *G. boninense* strain FA5017 with 99% similarity.

### Germinated Seeds

Ergosterol was detected in different concentrations in all inoculated germinated oil palm seeds and were absent in the un-inoculated germinated seeds (healthy samples). These concentrations apply for all the sampling periods determined from hour 6 to hour 168 after inoculation. Ergosterol was detected by visual evaluation image of TLC plates under UV-light in all inoculated seedlings (Fig. S1).

**Figure 2:**
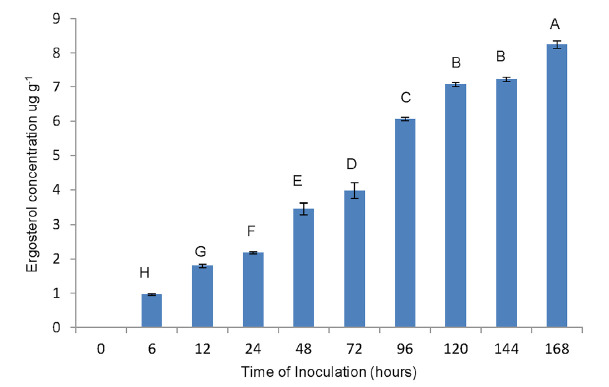
Ergosterol concentration of the germinated seeds in un-inoculated and inoculated germinated seeds. No ergosterol was detected in un-inoculated germinated seeds. Bars represent SE (standard error) of triplicate determinations.

The *R_f_* values of all samples were similar with that ergosterol standard spot with the value of 0.68. However, the ergosterol spot intensity under UV-light was faint and no detectable differences in inoculated seedlings at 6, 12, 24, 48, 72, and 96 hours after inoculation. However, the spot intensity increased gradually at hours 120, 144 and 168 after inoculation. High performance liquid chromatography analysis showed that ergosterol concentration increased with the increase of inoculation period (Fig. 2).

**Figure 3:**
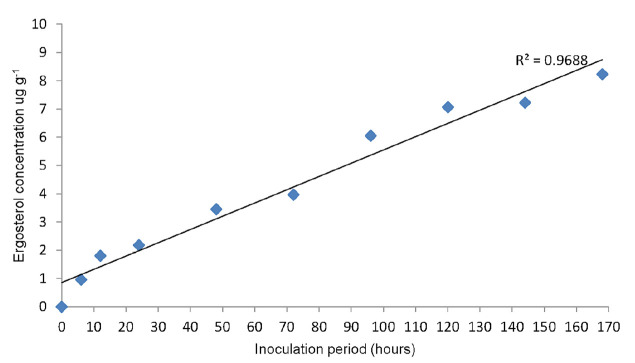
Relationship of inoculation period and ergosterol concentration of oil palm germinated seeds.

Ergosterol was detected as early as hour 6 after inoculation. Ergosterol concentration was significantly different from each sampling time from hour 6 to hour 168 after inoculation, however hour 120 and hour 144 did not show any significant differences after inoculation. The highest ergosterol concentration was 8.24 μg g^−1^ at hour 168 after inoculation with *G. boninense* culture, while the lowest ergosterol concentration was 0.96 μg g^−1^ on hour 6 after inoculation. A good correlation was observed between the inoculation period and ergosterol concentration (*R*^2^ = 0.97) (Fig. 3).

Nucleic acid extracted using CTAB method from oil palm germinated seeds was further confirmed using molecular identification. Deoxyribonucleic acid (DNA) amplification with *Ganoderma* specific primer Gan1 and Gan2 were analysed with 1.5% agarose followed by ethidium bromide staining showed visible band for the inoculated germinated seeds on the expected region at 150–200 bp, while no visible band was observed for the un-inoculated germinated seed (Fig. S2). Gene bank database confirmed the samples were highly similar to *G. boninense* strain FA5017 with 99% similarity.

**Figure 4:**
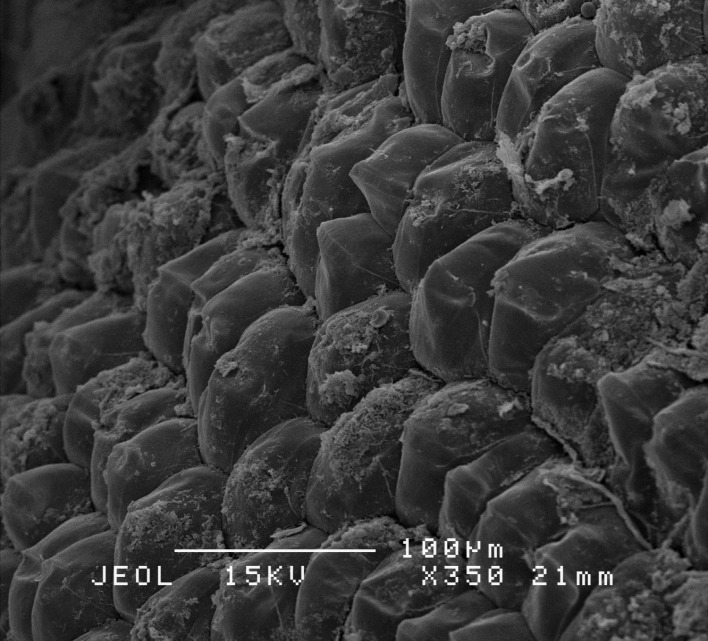

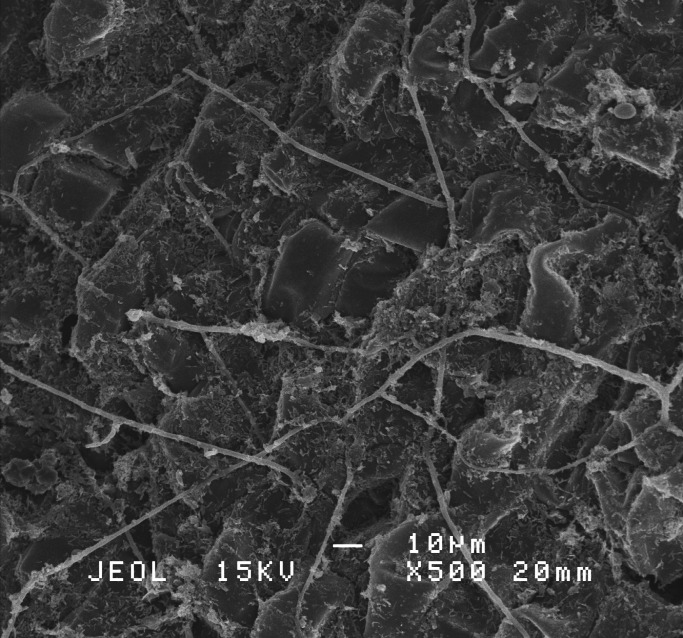

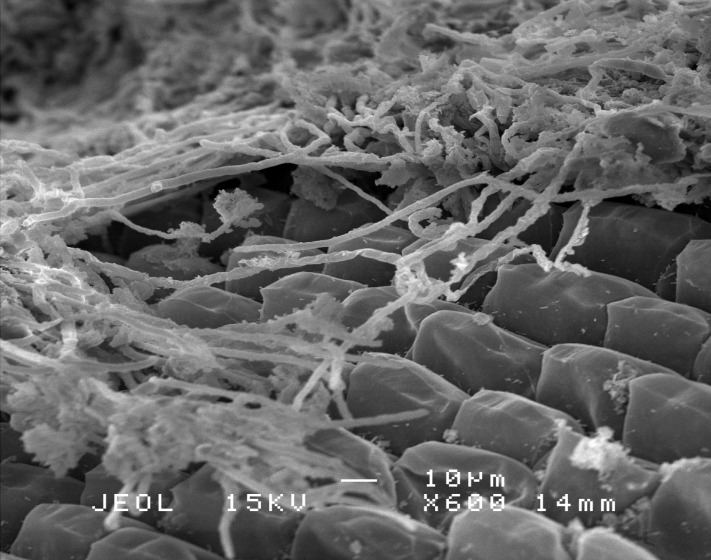

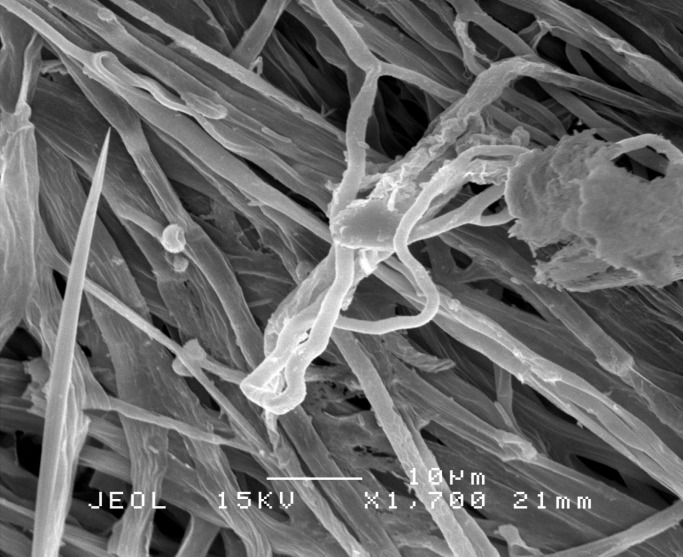
Comparison between inoculated and non-inoculated root: (A) non-inoculated root, (B) 6 h after inoculation, (C) 24 h after inoculation, (D) 48 h after inoculation with *Ganoderma* culture.

These results were further confirmed by SEM. Whereby, SEM demonstrated there were no hyphae of *Ganoderma* sp. detected on the root (Fig. 4A). In contrast, inoculated roots showed hyphae volumes increased with the increasing time of the inoculation period (Figs. 4B, C and D). The hyphae volume was very low at hour 6 after inoculation, however the hyphae started to colonise the roots at hour 24 after inoculation, and fully colonised the roots at hour 48 after inoculation.

Ergosterol was detected as early as hour 6 after inoculation. Ergosterol concentration was significantly different from each sampling time from hour 6 to hour 168 after inoculation, however hour 120 and hour 144 did not show any significant differences after inoculation. The highest ergosterol concentration was 8.24 ug g^−1^ at hour 168 after inoculation with *G. boninense* culture, while the lowest ergosterol concentration was 0.96 ug g^−1^ on hour 6 after inoculation. A good correlation was observed between the inoculation period and ergosterol concentration (*R*^2^ = 0.97) (Fig. 3).

### Oil Palm Seedlings

Ergosterol was detected in different concentrations in all inoculated oil palm seedling roots from the samplings of day 3 to week 28. Ergosterol was also detected via images of TLC plates under UV-light in all inoculated seedlings (Fig. S3). The R*_f_* values of all samples were similar to that ergosterol standard spot with the value of 0.68cm. However, the ergosterol spot intensity under UVlight was faint and no detectable differences was found in inoculated seedlings at day 3, 7, 14, and week 4. Nonetheless, the spot intensity increased steadily from week 8 to week 28.

**Figure 5:**
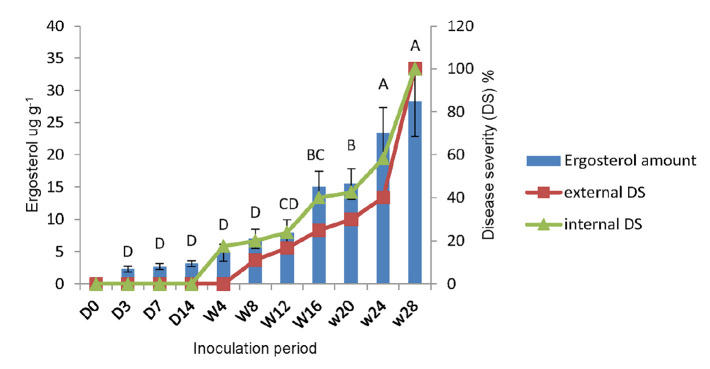
Ergosterol concentration and disease severity percentage in inoculated seedlings. Bars represent SE (standard error) of triplicate determinations. (D = day; W = week; DS = disease severity).

High performance liquid chromatography analysis showed ergosterol concentration increased parallel with the in internal and external disease severity from week 4 to week 28 (Fig. 5).

However, from day 3, 7 and 14, HPLC analysis quantified small amount of ergosterol from the samples, although there were no internal and external disease symptoms observed. The highest ergosterol concentration was 28.22 μg g^−1^ on week 28 with 100% external and internal disease severity where most of the palms were already dead while the lowest ergosterol concentration was 1.04 μg g^−1^ on day 3 with no observation of external and internal disease severity. A parallel correlation was observed between the internal disease severity with the ergosterol concentration (*R*^2^ = 0.95) and external disease severity with the ergosterol concentration (R^2^ = 0.85) (Figs. S4 and S5) respectively.

The oil palm seedling inoculated with *G. boninense* showed the presence of mycelium on the surface of the roots causing lesion and rotting to the roots, and the damages were observed increasing from day 3 to week 28 after inoculation (Fig. S6). In addition, the infected palm showed a sign of stunted growth (Fig. S7) and lesion of bole (Fig. S8) when compared to the healthy seedlings. These symptoms can be observed clearly as the infection progresses from day 3 to week 28 after inoculation. From this study, internal disease severity can be detected a month after inoculation followed by external disease severity which can be observed two month after the inoculation.

### Detection of Ergosterol from Infected Field Palm

**Figure 6:**
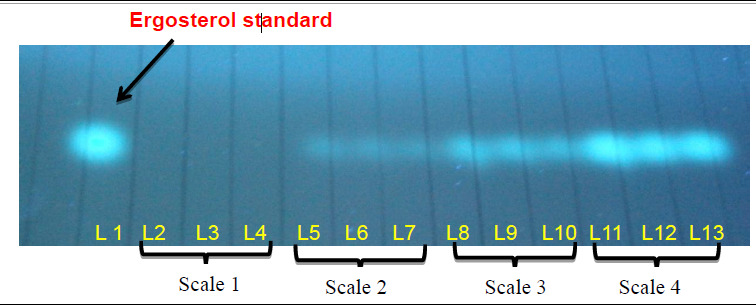
TLC analysis of ergosterol from field palms based on different level of external BSR infection. Lane 1: Ergosterol standard, Lane 2–4: Scale 1 palms, Lane 5–7: Scale 2 palms, Lane 8–10: Scale 3 palms, Lane 11–13: Scale 4 palms. (Scale 1 = Palms apparently normal and free from disease; Scale 2 = Asymptomatic neighbouring palms with the infected palms; Scale 3 = Palms with the presence of basidiomata at base of trunk; Scale 4 = Appearance of foliar symptoms and presence of basidiomata at base of trunk).

Ergosterol was detected from all sample categorised in scale 2, 3 and 4 and absent from the scale 1 palms when analysed via visual evaluation of images with RP-18 Silica coated TLC plates (Merck) in UV-light based on the ergosterol standard spot. (Fig. 6). The *R_f_* value of all detected samples were 0.68 (n-hexane: ethyl acetate) which is similar to *R_f_* value of the ergosterol standard spot. The intensity of the spot increased in samples from scale 4, where the disease symptoms appeared as the appearance of foliar symptoms and presence of basidiomata at base of trunk.

**Figure 7:**
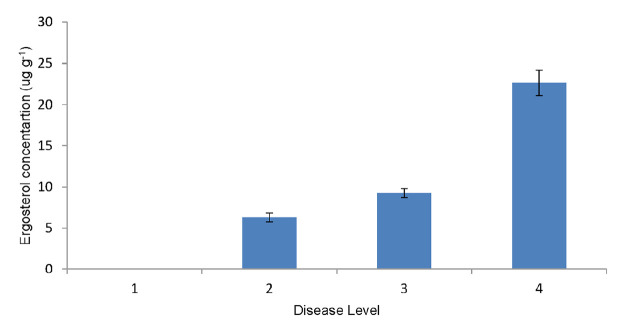
Average ergosterol concentration (μg g^−1^) compared to degree of BSR external symptoms (Scale 1 = Palms apparently normal and free from disease; Scale 2 = Asymptomatic neighbouring palms with the infected palms; Scale 3 = Palms with the presence of basidiomata at base of trunk; Scale 4 = appearance of foliar symptoms and presence of basidiomata at base of trunk). Bars represent ± SE (standard error) of triplicate determination.

In the HPLC analysis, ergosterol was identified by comparison of the retention time of ergosterol standard in all field palm samples. The ergosterol peak was well resolved and eluted at average of 7 min and the UV absorbance spectrum of ergosterol was clearly detected at 282 nm. The average concentration of ergosterol for scale 2, 3 and 4 were 6.31, 9.27 and 22.65 μg g^−1^, respectively (Fig. 7). The ergosterol concentration varied from each infected palm with the concentration (Scale 2, 1–15) ranging from 3.59–11.03 μg g^−1^, (Scale 3, 1–15) 5.95–14.4 μg g^−1,^ and (Scale 4, 1–15) 17.11–39.33 μg g^−1^.

## DISCUSSION

*G. boninense* ergosterol concentration in artificially inoculated oil palm seedlings increased directly with the increase of inoculation period from day 3 to week 28. This finding was supported by Mohd Aswad *et al.* (2011) and Toh Choon *et al.* (2011). This research also carried out an experiment on oil palm germinated seeds to study the relationship between inoculation time and ergosterol concentration and also to observe the earliest ergosterol detected prior inoculation with *Ganoderma*. From the results, ergosterol was detected as early as six hours after inoculation which means that ergosterol can be detected once the fungus start to colonise the root; thus, indicates that *G. boninense* mycelial mass colonising the oil palm roots increases in abundant as a sign of disease progression following the period of inoculation. Xue *et al.* (2006) conducted a study on the correlation between ergosterol content of soybean fungal pathogens; *Diaporthe phaseolorum*, causal agent of Phomop sis seed decay, and *Cercospora kikuchii*, causal agent of leaf blight and purple seed stain and biomass of these pathogens on the host plant. The findings of the present study was also inline with Xue *et al.* (2006) whom reported that biomass was manipulated by the varying incubation period and resulted in the linearity correlation between fungal dry mass and ergosterol content.

A strong relationship between *G. boninense*’s ergosterol concentration and oil palm disease severity was recorded both internally and externally in artificially inoculated oil palm seedlings. The current findings were supported by a study conducted by Frey *et al.* (1992) which reported that concentration of ergosterol in infected roots differ significantly from control plants. In addition, Mohd Aswad *et al.* (2011) reported that ergosterol concentration detected from inoculated oil palm seedlings increased significantly with the increased degree of root infection. In the present study, the higest ergosterol concentation detected was 28.22 μg g^−1^ on week 28 with 100% root infection, while the lowest ergosterol concentration was 1.04 μg g^−1^ on day 3 after inoculation with no visible root infection. Detection of ergosterol as early as day 3 after inoculation indicated rapid disease establishment by *G. boninense* in the root tissues. In support to this finding, Nusaibah *et al.* (2016) reported that symptoms of *Ganoderma* infection on artificialy infected oil palm seedling roots were visible via SEM,Transmission Electron Microscopy (TEM) and plant defense response against *Ganoderma* attack via plant metabolites were also identified after 24 hours of inculation period. In contrary, Mohd Aswad *et al.* (2011) managed to detect ergosterol via HPLC in *G. boninense* inoculated oil palm roots after 3 weeks. This result was in contrast with the present study, and this could be due to the sample preparation before extraction. Whereby in the present study, roots were not washed or surface sterilised prior to ergosterol extraction, which enabled ergosterol detection on the colonised root surface tissues. Ergosterol was found in high concentrations as a fungal cell wall component (Gessner 2005). In this experiment, the soil was sterilised prior to artificial inoculation steps to confirm that the soil used is free from other fungal contaminants. The results also demonstrated that external disease severity can be observed as early as week 8 after inoculation. Similarly, a study by Idris *et al.* (2006) reported that the foliar symptoms can be observed as early as two months after root inoculation in germinated seedlings.

For the field palm tissue samples, ergosterol was not detected in palms categorised in scale 1 which showed no visible symptoms. However, HPLC quantifies a small amount of ergosterol from scale 2 palms described as asymptomatic palms adjacent to the infected palms. Asymptomatic palms does not mean that the palm was free from disease. Mazliham *et al.* (2007) reported that visible symptoms of *Ganoderma* infection occurs at later stages of infection. Therefore from this finding, MAE method can be used as an early detection method of *Ganoderma* infection. HPLC quantified the highest ergosterol concentration in oil palm samples from scale 4 which exhibited the most severe symptoms. No significant differences were detected in ergosterol concentrations from scale 2 and 3 of infection. The average ergosterol concentration quantified by HPLC for scale 2, scale 3 were, 6.31 and 9.27 μg g^−1^ respectively. However, palms with severe disease level in scale 4 resulted in highest ergosterol concentration with 22.65 μg g^−1^ which was significantly different from palms from scale 1, 2 and 3. Basal stem rot disease was reported as a white wood rotting process involving growth of the fungus within the oil palm tissues vialignin and cellulose biodegradation (Paterson 2007b). Cellulose may be degraded readily by many fungi to gain energy, whereas lignin is a much more recalcitrant organic polymers that requires more energy for degradation purpose. *Ganodema boninense* was identified as a white rot fungus (Paterson 2007b) which can fully degrade the lignin component with progression of the disease infection. By doing so, the biomass grew with a consequent increase in ergosterol, lignocellulotic enzyme and a weakening of the oil palm. Hence, detection of ergosterol could quantify the amount of growth which is related to the damage of oil palm.

In this study, no ergosterol was detected in non-inoculated and healthy palms which indicates that the ergosterol detected in the disease palm is from *G. boninense.* The non-inoculated germinated seeds and seedlings grown under the same condition with inoculated samples showed no ergosterol detection when assesed using TLC and HPLC. This result indicates that healthy oil palm seedlings do not produce ergosterol as sterol compound. In addition, Zaiton *et al.* (2008) reported that most common endophytic microorganism found in healthy roots of symptomless palms were endophytic bacteria, and bacteria do not produce ergosterol. Therefore, ergosterol could be a useful biochemical marker in detection of BSR disease in oil palm.

In the field palms, the sampling technique utilised played an important role to avoid contamination from other fungi. The oil palm trunk were drilled 0.5–1.0 m from the base of the palm to avoid contamination of microbes from the soil. Besides that, the driller used also was sterilize with 70% ethanol. To eleminate the fungus from the surface of oil palm trunk, the trunks were first drilled into 1–2 cm depth, and the driller was sterilised again before drilling further into the trunk. In this present study, extraction of the oil palm tissues were conducted within a week after the sampling period to avoid any contamination or decomposes of the samples. Fresh samples have to be used to obtain reproducible ergosterol data, and try not to expose the extracts to direct sunlight for prolonged period. Frey *et al.* (1992) reported that preliminary study conducted have shown that ergosterol decomposes when the root samples are dried or exposed to the ultraviolet light. These findings were similar to that reported by Newell *et al.* (1988) where ergosterol also degrades during freezing or lyophilisation process.

Results obtained from SEM showed the hyphae colonisation on the root samples inoculated with the *G. boninense*. As the fungal biomass increase, the hyphae colonisation also increases with increased period of inoculation. Therefore, the ergosterol concentration increase with the increasing inoculation period of time. Rees *et al.* (2009) examined the *G. boninense* mode of infection using light microscopy and TEM and reported that root infection occurred consequence to firm attachment of *Ganoderma* hyphae to the root surface either localised to the initial point of contact or sometimes the fungus completely colonises the root at the point of contact. Mille-Lindblom *et al.* (2004) reported the ergosterol method as a major advance in the estimation of fungal biomass. Larsen *et al.* (2004) also stated that ergosterol is the principal membrane sterol of most fungi and commonly used for estimating living fungal biomass.

In germinated seeds, the results were further confirmed using molecular detection. Polymerase chain reaction technique was employed to identify *Ganoderma* species (Moncalvo *et al*. 1995; Idris *et al*. 2003; Chong *et al*. 2011). PCR product analysed resulted in the amplification fragment between 150–200 bp. Nevertheless, Utomo and Niepold (2000) conducted PCR using Gan1 and Gan2 on diseased oil palm roots samples and obtained amplified fragment size of 167 bp. Polymerase chain reaction amplification using *Ganoderma* specific primer Gan1 and Gan2 identified the pathogen as *G. boninense* with 99% similarity when BLASTn analysis performed on GenBank database. From the results of molecular identification, we could confirm the presence of *G. boninense* in the inoculated samples. Therefore, it was proven that the ergosterol detected in the oil palm root samples were from *G*. *boninense* fungal pathogen.

This present study also showed that the result of TLC were similar to the results obtained from quantification of ergosterol by using HPLC. For the germinated seeds and seedlings, the spot intensity of the TLC detection increase with the increase of inoculation period. HPLC also quantified the amount of ergosterol increased with the increasing inoculation period of time. In the field palm tissues, the spot intensity of the ergosterol detected in TLC showed the highest intensity for samples in scale 4. However, no significant difference in the spot intensity detected in palms from scale 2 and scale 3 of infection. This result correlates with the results obtain with HPLC quantification where the highest ergosterol concentration produced were palms in scale 4 which was significantly different from palms in scale 2 and 3, while for palms in scale 2 and 3 showed no significant difference statistically.

## CONCLUSION

Therefore, from this result, we could conclude that TLC analysis correlated well with the HPLC quantification. Thus, TLC analysis could be used for detection of ergosterol on the field palms as it is a more convenient and can be carried out on site besides suitable for large field survey during the census. Seitz *et al.* (1977) also suggested that preparative TLC and spectrophotometry could be used to estimate ergosterol if HPLC equipment is not available. Furthermore, according to Naewbanij *et al*. (1984), TLC may detect ergosterol as low as 1 μg g^−1^. In addition, ergosterol concentration also demonstrated a positive relationship between *Ganoderma* biomass and BSR development in artificially inoculated germinated seeds and seedlings. Moreover, ergosterol may be detected as early as six hours and three days after inoculation on germinated seeds and seedlings respectively using MAE method. In addition, the comparison between different extraction method was conducted and has been reported by Muniroh *et al.* (2014) that showed MAE as the most efficient compared to NAE and USE methods. Therefore, the use of MAE method in extracting ergosterol is suitable for the detection of BSR disease in field palms.

## Supplementary Data

Figure S1 Ergosterol detection from uninoculated and inoculated germinated seeds by TLC. Ergosterol standard: Lane 1; Uninoculated seedlings: Lane 2–4; Inoculated seedlings (6, 12, 24, 48, 72, 96, 120, 144 and 168 hrs): Lane 5–13.

Figure S2 PCR amplification of inoculated and non-inoculated germinated seeds. L1, non-inoculated germinated seed; L2–L10, inoculated germinated seeds (6, 12, 24, 48, 72, 96, 120, 144, 168 hours); M: 100bp Marker.

Figure S3 Ergosterol detection from uninoculated and inoculated oil palm seedlings by TLC. Ergosterol standard: Lane 1; Uninoculated seedlings: Lane 3–6; Inoculated seedlings (day 3, 7, 14 week 4, 12, 16, 20, 24, 28): Lane 7–16.

Figure S4 Relationship between ergosterol concentrations detected via HPLC and internal disease severity of oil palm seedling from *G. boninense.*

Figure S5 Relationship between ergosterol concentrations quantified via HPLC and external disease severity of oil palm seedling from *G. boninense.*

Figure S6 Comparison between healthy root and damage root: (A) Healthy root; (B) Arrow shows root with the presence of white mycelium 3 days after inoculation; (C) Arrow shows rotting root; (D) Arrow shows lesion of root 16 weeks after inoculation.

Figure S7 Comparison between healthy palm (left) and infected palm (right) at 16 weeks after inoculation. Infected palms show stunted growth.

Figure S8 Comparison between (A) healthy bole and (B) infected bole 20 weeks after inoculation.

## Figures and Tables

**Table 1:** Disease severity scale.

Scale	Internal DS (root lesion)	External DS (foliar symptoms)
0	Healthy: no damage	Healthy
1	< 10% rotting of roots; bole lesion	Yellowing of lower leaves and formation of rhizomorph at base of bole
2	10%–20% rotting of roots; bole lesion	Necrosis of lower leaves and emergence of button-like sporophore at base of bole
3	20%–50% rotting of roots; bole lesion	More than 50% necrosis of leaves and production of sporophore at base of bole
4	> 50% rotting of roots; bole lesion	Total necrosis and production of basidiomata at base of bole

**Table 2:** Description of external BSR symptoms based on disease category level.

Disease scale	Signs and symptoms
Scale 1	Palms apparently normal and free from disease
Scale 2	Asymptomatic neighbouring palms with the infected palms
Scale 3	Palms with the presence of basidiomata at base of trunk
Scale 4	Appearance of foliar symptoms and presence of basidiomata at base of trunk
