# Supplementary figures and images for: Relationship between Ganoderma Ergosterol Concentration and Basal Stem Rot Disease Progress on Elaeis guineensis
Source: Trop Life Sci Res. 2020 Apr 7;31(1):19–43. doi: 10.21315/tlsr2020.31.1.2 (PMC7485531; doi:10.21315/tlsr2020.31.1.2)

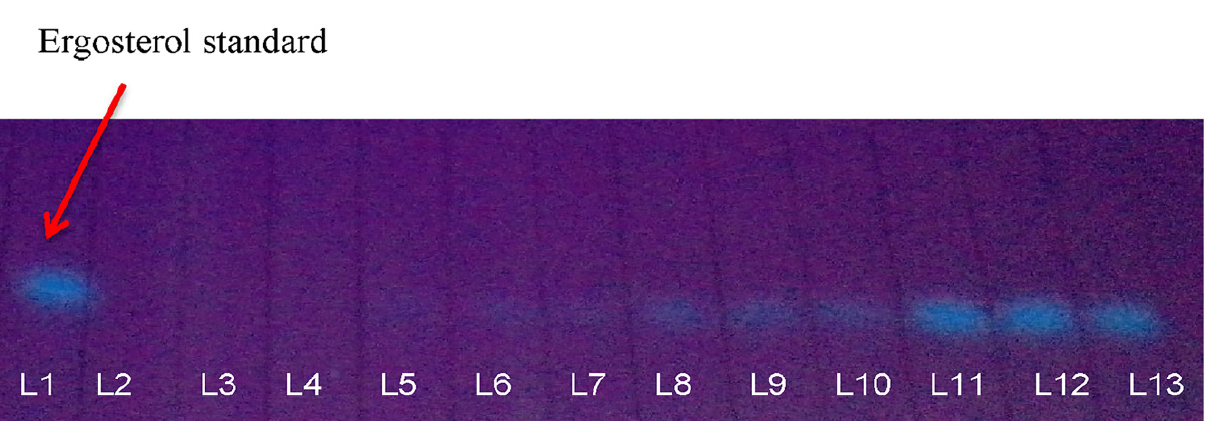

Supplement: Figure S1 — Ergosterol detection from uninoculated and inoculated germinated seeds by TLC. Ergosterol standard: Lane 1; Uninoculated seedlings: Lane 2–4; Inoculated seedlings (6, 12, 24, 48, 72, 96, 120, 144 and 168 hrs): Lane 5–13. [file TLSR-31-1-19-g008-S1.tif]

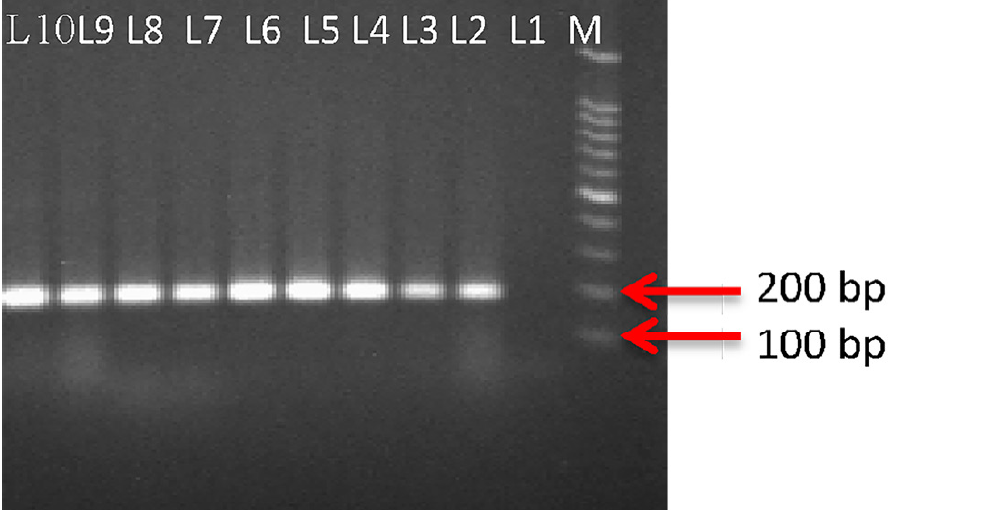

Supplement: Figure S2 — PCR amplification of inoculated and non-inoculated germinated seeds. L1, non-inoculated germinated seed; L2–L10, inoculated germinated seeds (6, 12, 24, 48, 72, 96, 120, 144, 168 hours); M: 100bp Marker. [file TLSR-31-1-19-g008-S2.tif]

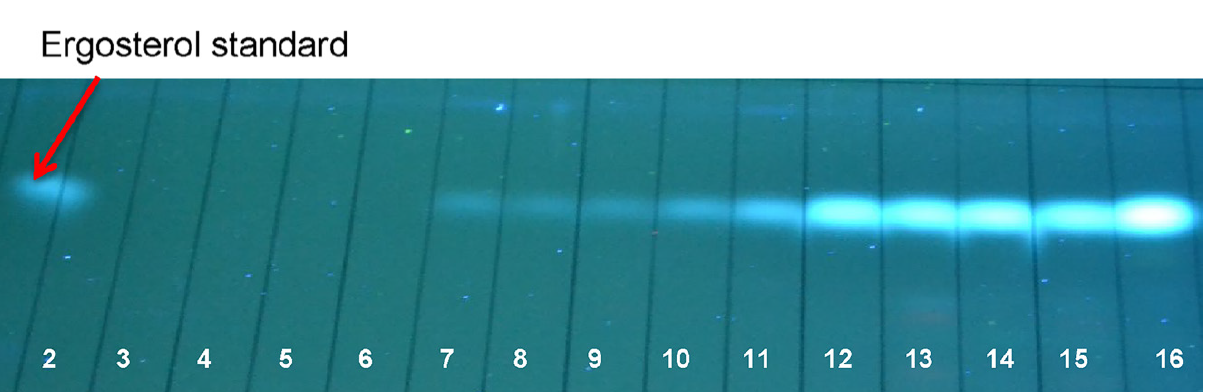

Supplement: Figure S3 — Ergosterol detection from uninoculated and inoculated oil palm seedlings by TLC. Ergosterol standard: Lane 1; Uninoculated seedlings: Lane 3–6; Inoculated seedlings (day 3, 7, 14 week 4, 12, 16, 20, 24, 28): Lane 7–16. [file TLSR-31-1-19-g008-S3.tif]

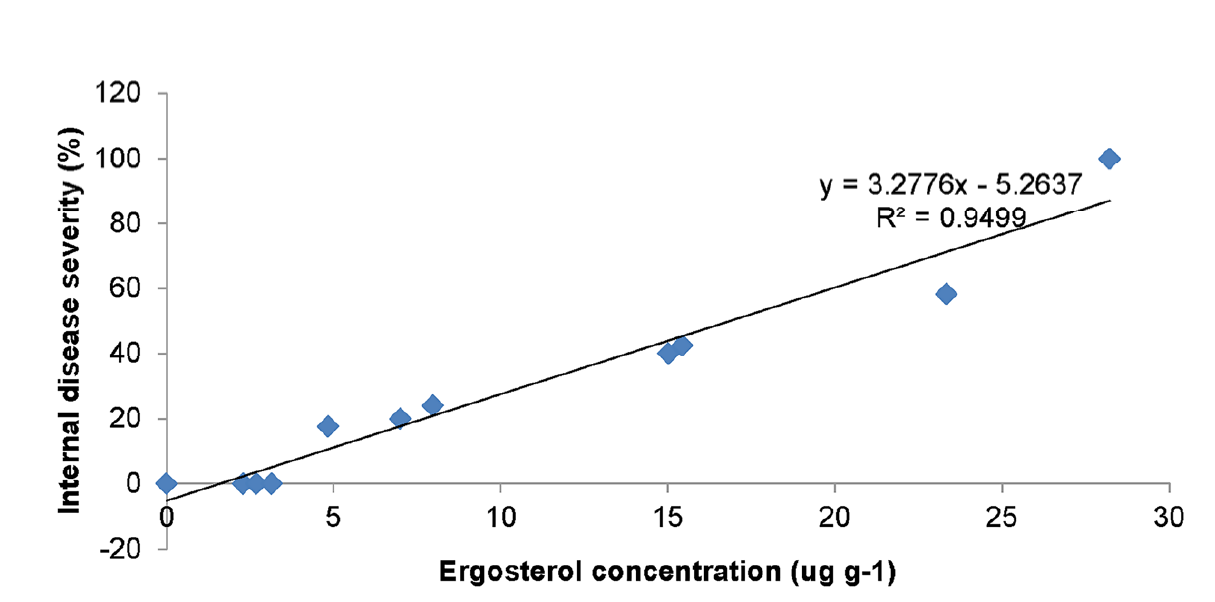

Supplement: Figure S4 — Relationship between ergosterol concentrations detected via HPLC and internal disease severity of oil palm seedling from G. boninense. [file TLSR-31-1-19-g008-S4.tif]

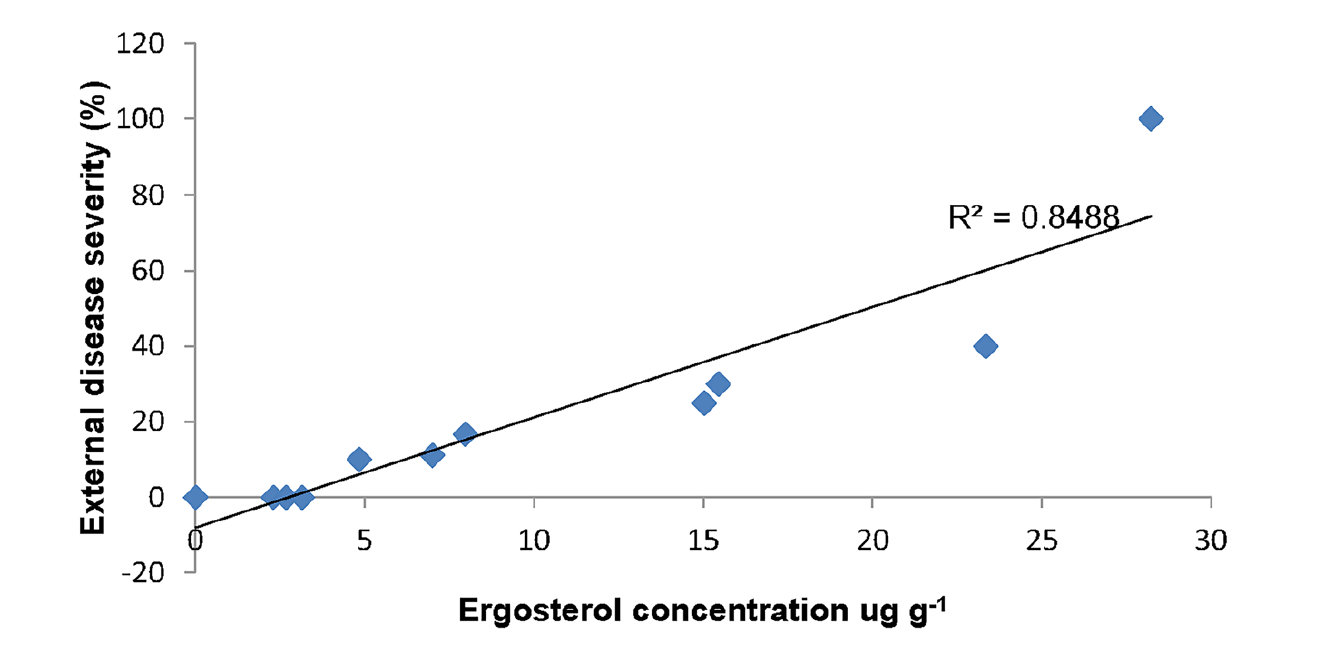

Supplement: Figure S5 — Relationship between ergosterol concentrations quantified via HPLC and external disease severity of oil palm seedling from G. boninense. [file TLSR-31-1-19-g008-S5.tif]

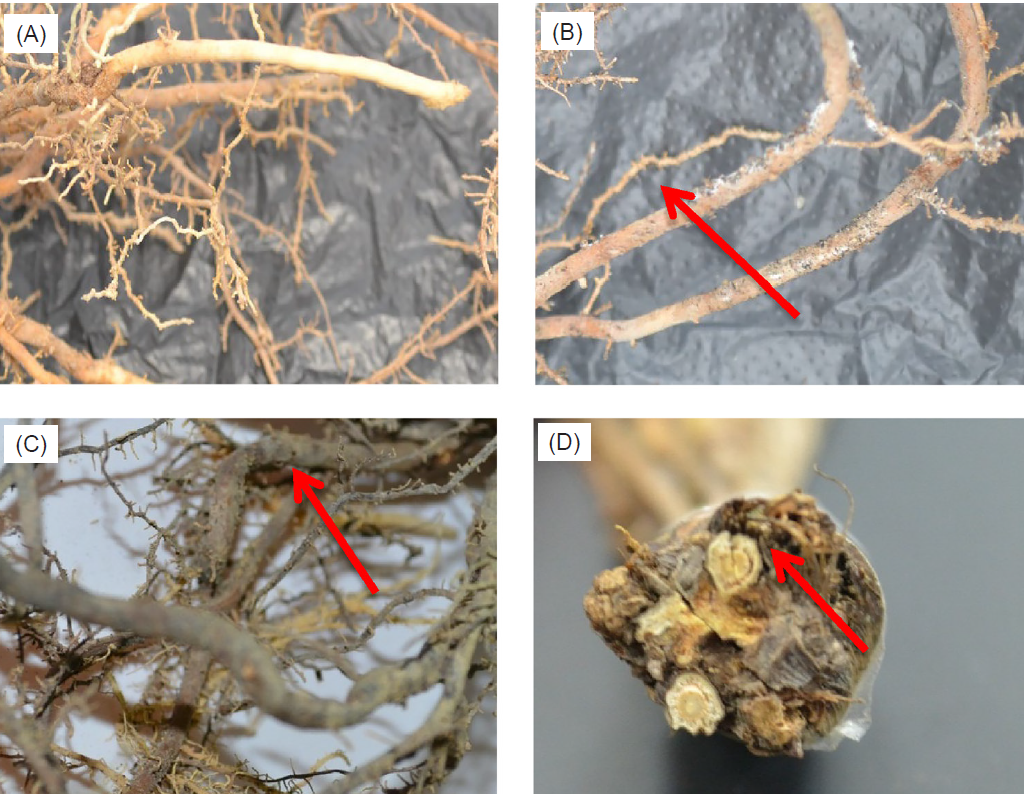

Supplement: Figure S6 — Comparison between healthy root and damage root: (A) Healthy root; (B) Arrow shows root with the presence of white mycelium 3 days after inoculation; (C) Arrow shows rotting root; (D) Arrow shows lesion of root 16 weeks after inoculation. [file TLSR-31-1-19-g008-S6.tif]

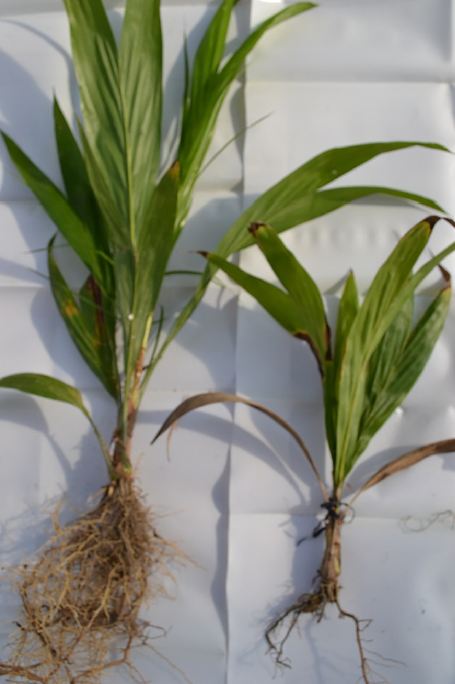

Supplement: Figure S7 — Comparison between healthy palm (left) and infected palm (right) at 16 weeks after inoculation. Infected palms show stunted growth. [file TLSR-31-1-19-g008-S7.tif]

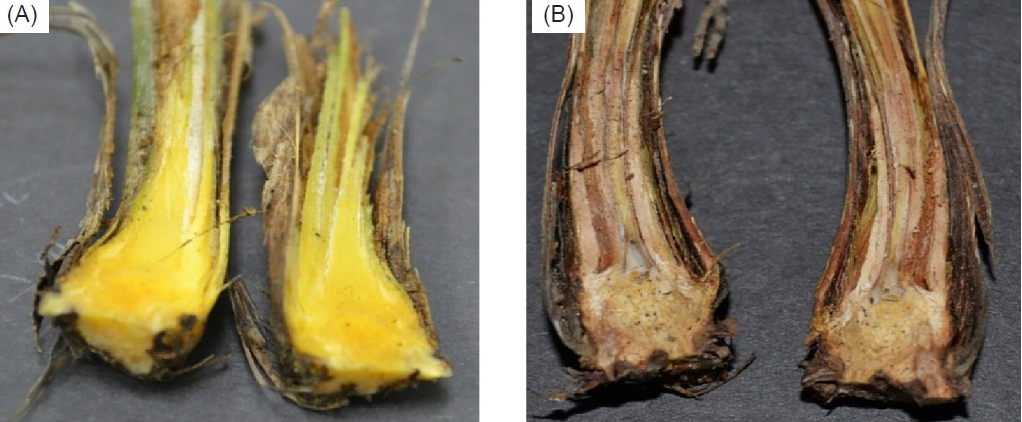

Supplement: Figure S8 — Comparison between (A) healthy bole and (B) infected bole 20 weeks after inoculation. [file TLSR-31-1-19-g008-S8.tif]
